# Paraneoplastic Pemphigus with Underlying Castleman's Disorder: A Rare Report with Literature Review

**DOI:** 10.7759/cureus.5022

**Published:** 2019-06-28

**Authors:** Syed Hamza Bin Waqar, Anosh Aslam Khan, Osama Mohiuddin, Aiman Rehan

**Affiliations:** 1 Internal Medicine, Civil Hospital Karachi, Dow University of Health Sciences, Karachi, PAK; 2 Internal Medicine, Dow University of Health Sciences, Karachi, PAK

**Keywords:** paraneoplastic pemphigus, pnp, castleman's disease, unicentric, polymorphous skin lesion, bullous disorder, ucd, ivig

## Abstract

Paraneoplastic pemphigus (PNP), also known as paraneoplastic autoimmune multisystem syndrome (PAMS), is an autoimmune blistering disorder of the skin associated with various hematological and nonhematological malignancies. In most of the cases, it can be a harbinger of a concealed benign or malignant neoplasm. We report the case of a 23-year-old female patient who presented to the dermatology consult service with a previously known diagnosis of refractory pemphigus vulgaris but she failed to reach remission for her oral and skin lesions on steroid and intravenous immunoglobulin (IVIG). She was later investigated for underlying malignancy as the concern of PNP was raised. She was found to be diagnosed with a pelvic mass which was found to be Castleman's disease. Our patient responded well to surgery and postoperative course of pulsed methylprednisolone and IVIG. Here, we discuss the diagnosis and clinical course of this unique case and strive to create awareness about PNP that can present as a refractory polymorphous blistering dermatological disorder and can hinder the diagnosis and management of patients.

## Introduction

Amongst various other autoimmune dermatological disorders, paraneoplastic pemphigus (PNP) holds a special place because of the heterogeneity and rarity of its presentation. Only a handful of cases have been reported to date. It presents as polymorphic mucocutaneous eruption recalcitrant to topical and systemic immunomodulating and suppressive therapies. It is characterized as a lethal disorder considering its risk of association with bronchiolitis obliterans, an irreversible obstructive pulmonary lesion. Therefore, timely diagnosis by physicians is crucial in preventing mortality that this disorder poses secondary to the variability of presentation of PNP. Mostly, PNP is associated with an underlying neoplasm that comes to light because of the refractoriness of the skin lesions to treatment. Castleman's disease is one such condition that shares ties with it. It is defined as a lymphoproliferative disorder and can produce autoantibodies that can lead to the condition, with remission only being possible once it is excluded from the system. Interestingly, despite the bad prognosis of PNP, cases associated with Castleman's syndrome are associated with an overall better outcome [1]. Herein presented is a case of a 23-year-old female who came to the dermatology consult service with refractory polymorphous skin lesions which were later diagnosed as PNP and the lack of any underlying neoplasm led to the diagnosis of Castleman's disorder. Timely diagnosis and appropriate treatment and follow-up resulted in remission of a deadly yet controllable paraneoplastic syndrome of skin.

## Case presentation

A 23-year-old female presented to the dermatology consult service with a false pre-diagnosis of pemphigus vulgaris for seven months. She had initially developed erythema and macules on the chest and shoulder which gradually transformed to vesicles and bullae with erythematous patches on the back and shoulders. The extension of bullae further progressed in number from being hardly a few to many with occasional oozing of serum and blood. The presentation of blisters coincided with the development of aphthous ulcers in the mouth which rapidly progressed to ulceration and severe stomatitis. There were no aggravating or relieving factors present. The patient also reported myalgias and fever since the beginning of the episodes with no accompanying pruritus. History was also significant for pain in the mouth which augmented on chewing and swallowing. She was followed over a period of four months but her oral lesions did not respond to topical immunosuppressants including steroids and steroid-sparing agents or to rituximab that was initially tried. The skin lesions, however, did respond to some degree with the reduction in bullae and erythema with the application of topical steroids and biobrane synthetic dressings. There was no hoarseness, dysphagia, dysuria, and dyspareunia. Her past medical, surgical, and family history was, however, insignificant.

On admission, she had skin lesions in various stages with erythema and bullae on the back and chest. She also had severe erosive mucositis and gingivitis with an extensive ulcer with a hematinic crust on the lateral aspect of the tongue. Bullae on slight compressional force dislodged the upper epithelium making the Nikolsky sign positive. The exam was also significant for bilateral conjunctival hyperemia but there were no erosion or ulcers. Her temperature was over 99°F with a pulse of 89 beats per minute. Blood pressure was 110/78 mmHg with a respiratory rate of 17 per minute. Her otorhinolaryngological, abdominal, nervous, pulmonary, and cardiac examination was unremarkable.

Due to the intractable nature of the lesions, a search for another entity similar to pemphigus, PNP was made. For making the definitive diagnosis, along with history, various laboratory methodologies were utilized. A 4-mm punch biopsy was taken from the erosive mucosa in the oral cavity which showed intralesional, suprabasal acantholysis, dyskeratosis, and dermo-epidermal junction interface dermatitis. A perilesional punch biopsy was taken for direct immunofluorescence (DIF) from the skin which highlighted intercellular deposition of immunoglobulin G (IgG) and complement C3. There was, however, minimal to absent deposition at the basement membrane. Enzyme-linked immunosorbent assays (ELISA) for antibodies against envoplakin and periplakin were utilized to effectively label the findings observed in DIF and lesional punch biopsy which turned out to be positive. ELISA for desmoglein-1 was also positive in the panel supporting the diagnosis. Immunoprecipitating studies and indirect immunofluorescence were, however, not performed due to unavailability and fulfillment of criteria required for diagnosis. Complete blood count indicated mild lymphocytosis. The other diagnostic studies done including erythrocyte sedimentation rate (ESR) and protein electrophoresis were within normal limits.

**Figure 1:**
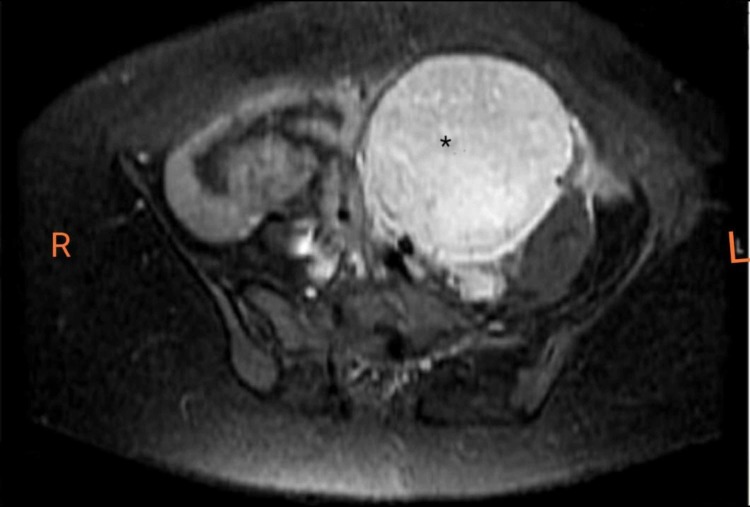
T1-Weighted MRI showing left-sided retro-peritoneal homogeneously enhancing, well-circumscribed mass (asterisk).

**Figure 2:**
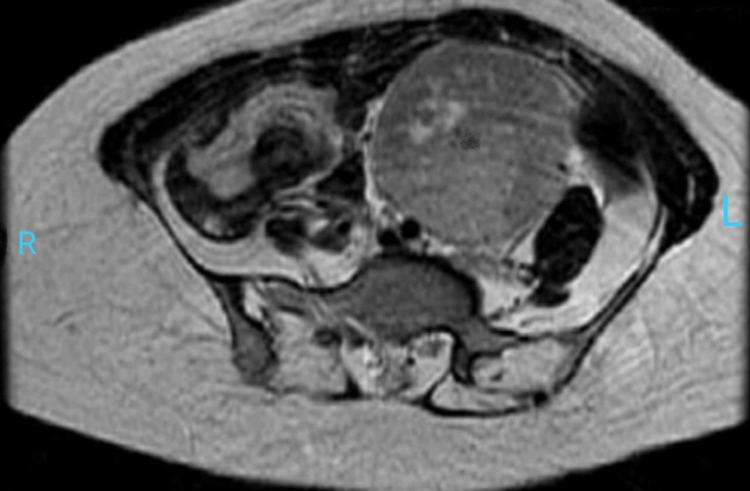
T2-Weighted MRI showing retro-peritoneal left-sided well-circumscribed, heterogeneous, and hyper-intense mass.

Radiological tests were also conducted to find the underlying culprit for PNP. CT chest was performed which was insignificant and showed no signs of any mass or bronchiolitis obliterans. CT abdomen and pelvis demonstrated a well-circumscribed mass of soft tissue attenuation in the pelvis with homogenous enhancement on contrast. MRI also demonstrated slight hyperintensity and heterogeneity on T2 imaging. There were some intra-lesional flow voids on T1 and T2 images which depicted high vascularity of the lesion with central linear hypo-intense septae (Figures 1-2).

Extensive workup included lactate dehydrogenase (LDH) which was 700 IU/L (normal: 150-300 IU/L); ELISA for human immunodeficiency virus (HIV) and human herpes virus-8 (HHV-8) was negative, rest of the investigations involving urine beta-2 microglobulin and serum light chain analysis were negative. This along with the histopathological support from biopsy made the diagnosis of unicentric Castleman's disease (UCD).

Aseptic measures were employed from the very first day of admission and antimicrobials were administered. The tumor was localized and excised after which a postoperative CT scan was done to look for any remaining active lymph node. LDH levels also fell back to normal in a week. Intravenous immunoglobulin (IVIG) was given pre- and post-operatively in the dosage of 2 g/kg with pulsed methylprednisolone therapy of 1 g/day infusion for three days. This resulted in remission of the mucocutaneous lesions. The patient maintained remission in follow-ups which showed resolution of the erosive mucositis and diffuse ulceration involving the mouth with no consequent use of the steroid. The patient was also closely followed-up with a pulmonologist for the possible development of bronchiolitis obliterans, an impending complication of UCD with PNP. Fortunately, the patient did not exhibit any signs or symptoms of developing this complication.

## Discussion

Paraneoplastic pemphigus is a rare and often fatal autoimmune blistering disorder with polymorphous skin lesions that can mimic pemphigus vulgaris and other dermatological conditions. It primarily causes blisters and ulcerated lesions in mucocutaneous areas and is known to herald benign and malignant neoplasms. The most commonly associated neoplasms are non-Hodgkin’s lymphoma, chronic lymphocytic leukemia, Castleman’s disease (CD), thymoma, sarcoma, and Waldenstrom's macroglobulinemia. Epidemiologically, adults with ages between 45 and 70 years are more prone to develop PNP. Moreover, children diagnosed with CD are also predisposed to it. Till today, a correlation on the basis of gender, ethnicity, and geography has not been established [1]. PNP presents with a spectrum of clinical features comprising refractory oral lesions, polymorphic cutaneous lesions ranging from erythema, vesicles, bullae, inflammatory papules and plaques with a positive Nikolsky sign, targetoid lesions to extensive desquamation, and pulmonary epithelial involvement, often leading to life-threatening bronchiolitis obliterans. Hence, a more inclusive term ‘Paraneoplastic Autoimmune Multiorgan Syndrome or PAMS’ has been coined [2]. The ulceration of the mucous membrane is not only limited to the oral cavity but can also involve the esophagus, stomach, duodenum, and other parts of the intestine [1].

In terms of etiopathogenesis, the tumor-induced dysregulated immune system produces autoantibodies against the plakin and cadherin family of proteins that cross-react with the epithelial antigens. These include 250- and 210-kilodalton (kDa) desmoplakins I and II (DSPI and DSPII), 230-kDa bullous pemphigoid antigen (BP230), 210-kDa envoplakin, 190-kDa periplakin (PP), 170-kDa protease inhibitor alpha2-macroglobulin-like-1 (A2ML1), 160- and 130-kDa desmogleins (Dsg) 1 and 3, 500-kDa plectin, plakophilin 3 and desmocollin 1 to 3. Additionally, cell-mediated immunity also contributes a significant part in the development of pathology. These characteristics demonstrate the immunological complexities of the disease. Therefore, several laboratory techniques and immunological studies have been introduced for the diagnosis of PNP. Under the light microscope, biopsy exhibits suprabasal acantholysis, keratinocyte necrosis, and lichenoid interface dermatitis with lymphocytic infiltrates. As histopathology may vary and intersect with multiple disorders, immunofluorescence studies are sought. Direct immunofluorescence microscopy shows a typical linear deposition pattern of immunoglobulin G (IgG) and/or complement C3 in basement membrane zones and intercellularly. It can also identify cytotoxic CD8+T cells that attack keratin layers, exhibiting intracellular staining at dermo-epidermal junctions. Indirect immunofluorescent studies, with murine’s urothelium as preferable substrate, demonstrate epithelial cell surface attachment of IgG and C3 [3]. Autoantibodies targeted towards plakins are the characteristic feature of PNP with the high specificity of envoplakin and periplakin about 99% and 96% respectively [4]. Most importantly, immunoprecipitation is considered as the gold standard diagnostic test for PNP, with the sensitivities of 95% for radioactive and 100% for nonradioactive immunoprecipitation tests. The specificity of both tests’ ranges between 86% and 100%. The combined immunoblot-indirect fluorescence test holds 100% sensitivity and specificity, acting as an alternate option to immunoprecipitation [3].

In 1993, Camisa and Helm put forward a modified algorithm for diagnosis of PNP which comprised a major and minor criteria. It was proposed that a patient should be considered a case of PNP if all three major criteria or two major and two minor criteria were fulfilled [5]. However, in 2002, Mimouni et al. presented four minimum criteria of the highest significance for the composite diagnosis [6]. These include:

1. Features of severe mucosal involvement with polymorphic cutaneous lesions

2. Detection of antiplakin autoantibodies

3. Histological findings of acantholysis or lichenoid or interface dermatitis

4. Presence of an underlying neoplasm especially lymphoproliferative malignancies.

Despite the advancement in the diagnostic approach, PNP still postulates a crucial challenge to physicians. It can be easily misdiagnosed as other dermatological conditions such as erythema multiforme, pemphigus vulgaris, bullous pemphigoid, cicatricial pemphigoid, lichen planus pemphigoids, radiation dermatitis, Steven-Johnson syndrome, toxic epidermal necrolysis, and even drug reactions, due to the similar presentation [7]. One of the critical differentiation markers between PNP and pemphigus foliaceus and vulgaris resides in the specificity of antigens. Here, the vitality of indirect immunofluorescence comes to play as the mouse’s urothelium does not express desmoglein (DSG) 1 and 3 which are the distinct antigens of pemphigus foliaceus and pemphigus vulgaris, respectively [8]. Due to the involvement of the mucous membrane in other similar diseases, few cases suggest a thorough otorhinolaryngological examination with a primary focus on the oral cavity as being pertinent and high yield [9].

Genetic studies have established that specific major histocompatibility complex (MHC) increases the susceptibility of certain autoimmune disorders. Likewise, in the case of PNP, preclusive studies indicate the specificity of human leukocyte antigen HLA-Cw*14 allele which remains statistically significant, whether the patient has CD or any other tumor [10].

Castleman’s disease is an uncommon lymphoproliferative disorder which has a relative concomitant occurrence of 18% with PNP [1]. It was first described in 1956 by Dr. Benjamin Castleman. Due to its rarity, research on various aspects of CD has progressed quite slowly over the years. Nevertheless, it is a heterogeneous disorder which is classified histopathologically into three variants which include a hyaline-vascular variant, plasmacytic variant, and a mixed cellular variant. Clinically, it is categorized as being ‘unicentric’ and ‘multicentric.’ The hyaline variant demonstrates sclerotic vessels penetrating radially in atrophic germinal center (so-called ‘lollipop’ appearance) and follicles surrounded by a broad mantle zone containing concentrically arranged lymphocytes (so-called ‘onion-skin’ appearance). The plasmacytic variant exhibits interfollicular plasmacytosis that shows positive immunostaining with patent sinus and intact architecture. The mixed cellular variant presents as an overlapping picture of the former variants with mature lymphocytes surrounding the worn off germinal center [11]. Unicentric CD (UCD) is more common between the ages of 30 and 40 and has slightly more prevalence among females. It can be asymptomatic or may present as painless lymphadenopathy, local mass effects, and anatomical symptoms. The most predominant location is within the chest while other areas like neck, abdomen, retroperitoneum, etc. can also be involved. Clinically, B-symptoms and deranged laboratory indicators are seldom noted and histologically, the hyaline vascular variant is a frequent presentation. Contrast tomography of chest, abdomen, and pelvis, serology for HIV and polymerase chain reaction (PCR) for HHV-8 should be performed as a part of the pre-treatment protocol and to differentiate it from multicentric CD [12].

As a standard therapy, surgical resection is pursued in almost every case of UCD with an impeccable curative rate. Recently, neo-adjuvant radiotherapy has also shown promising results for irresectable cases [13]. The literature review suggests the disease-free survival rate at three years to be 90%, however, PNP along with its complication especially bronchiolitis obliterans is considered as independent risk factors for poor prognosis of a condition as benign as UCD [11]. It is responsible for the mortality rate of 90% in the first year [14-15]. Hence, treatment of PNP requires crucial attention. Frew et al. devised a management plan for better outcomes of patients that began foremost with the stabilization of vital parameters of patients, followed by an assessment of any associated malignancy, confirming the diagnosis of PNP, and finally eradicating the triggering tumor and initiating treatment strategies. The normalization of vital parameters is considered as a single best preventive factor of death in these patients [16]. The first-line treatment is high dose corticosteroid (0.5-1.0 mg/kg), which dramatically improves cutaneous lesions but has a minimal effect on the mucosal lesions. This is also true for the steroid-sparing and immunosuppressant drugs which are used with glucocorticoid [1]. As a large number of autoantibodies can be spilled into the bloodstream during tumor excision surgery, complete resection of the tumor is the preferred approach with blockade of blood flow and gentle handling of neoplastic tissue. Moreover, the regimen of pulse methylprednisolone with intravenous immunoglobulin is being used before, during, and after surgery for significant improvement. It also leads to a prominent reduction in mortality caused specifically by bronchiolitis obliterans [11, 17]. The apt management of complications begins with the usage of systemic antimicrobial drugs to prevent sepsis as the skin integrity is marred in PNP and there is also a concomitant use of immunosuppressants. The judicious use of potent analgesics should also be provided for the comfort of the patient [18]. In light of a high mortality rate and resistance to multiple therapies, it is pivotal to follow these patients so as to prevent future complications and recurrence.

## Conclusions

Paraneoplastic is a rare and unusual autoimmune blistering disorder that can prove fatal if not diagnosed and differentiated from some relatively common skin conditions like pemphigus vulgaris. Given the queer presentation of recalcitrant erosive mucocutaneous lesions and refractoriness to immunosuppressant therapies, underlying search for malignancy or benign neoplasm contributing to the auto-antibodies should be made once the diagnosis of PNP, is itself made potentially clear, as can be seen in this case. Diseases like Castleman's syndrome if discovered amidst PNP survey, should be considered a blessing in disguise given its relative good prognosis and remission rates as compared to other neoplastic disorders.
